# Chloroform Anæsthesia Dangerous to Meat-Eaters

**Published:** 1896-06

**Authors:** 


### Chloroform Anæsthesia Dangerous to Meat-Eaters.

Some time ago Dr. Lauder Brunton called attention to the fact that death from chloroform anæsthesia is probably due, not to the chloroform itself, but to the fact that chloroform arrests the elimination of tissue poisons, and that death is directly the result of the action of these poisons rather than of the chloroform. Dr. Brunton cited the fact that death from chloroform anæsthesia is very rare in India, while it is becoming more and more common in England, which fact he attributes to the increasing use of meat as an article of diet in Great Britain.

Chloroform has long been a popular anæsthetic in Edinburgh, but recently deaths from its use in that city have been very frequent. It is also noticed that gout is becoming very common. Both these circumstances are doubtless due to the increased consumption of meat resulting from the large importation of low-priced refrigerator meat.—*Modern Medicine.*

